# Modifications to the nutrition for sport knowledge questionnaire (NSQK) and abridged nutrition for sport knowledge questionnaire (ANSKQ)

**DOI:** 10.1186/s12970-019-0293-8

**Published:** 2019-06-28

**Authors:** Gina Louise Trakman, Freddy Brown, Adrienne Forsyth, Regina Belski

**Affiliations:** 1 Department of Rehabilitation, Nutrition and Sport, School of Allied Health, College of Science, Health and Engineering, La Trobe University, Melbourne, 3086 Australia; 2 Faculty of health and life sciences, Coventry University, Coventry, England CV1 2TU UK; 3 Department of Health Professions, School of Health Sciences, Faculty of Health, Arts and Design, Swinburne University of Technology, Hawthorn, 3122 Australia; 4 English Institute of Sport, The Manchester Institute of Health and Performance, Manchester, M11 3BS UK

**Keywords:** Athlete, Nutrition knowledge, Questionnaire

## Abstract

New evidence and feedback from colleagues have led to modifications to the Nutrition for Sport Knowledge Questionnaire (NSKQ) and Abridged Nutrition for Sport Knowledge Questionnaire (ANSKQ). The changes predominately affect questions regarding the nutrient content of foods, protein recommendations and the legality of supplements. Some English language edits have also been made.

## Main text

In 2015–2017 *Trakman* et al. developed the Nutrition for Sport Knowledge Questionnaire (NSKQ) [1] and the Abridged Nutrition for Sport Knowledge Questionnaire (ANSKQ) [2]. The NSKQ and ANSKQ been used to assess Australian athletes’ nutrition knowledge [2–4]. The authors have received over 135 requests from researchers and professionals interested in utilising the tools. International researchers have translated the NSKQ into German, Italian, Swedish, Turkish, Chinese and Malay. Since the development of the NSKQ and ANSKQ, the International Society of Sports Nutrition (ISSN) published new consensus recommendations on protein [5, 6] and World Anti-Doping Agency (WADA) changed the legality of glycerol [7]. Moreover, administration of the tool and feedback from colleagues (in particular, Freddy Brown) has led to reflections regarding areas for improvement. The key changes are outlined below. Item numbers refer to the NSKQ and where relevant, these changes have also been applied to the ANSKQ.

Items modified due to lack of clarity:

- ***Item 2.5*** on high/low fat foods was based on The Australian Dietary Guidelines, which recommend patients choose foods with less than 10% fat (or 15% for cheese). However, in most countries, labelling laws [8, 9] state that foods can only be classed as low fat if they have less than 3% fat. Thus, classing foods such as cottage cheese (fat content approximately 1–8%) as low fat could be confusing. We replaced ‘cottage cheese’ with ‘cheddar cheese’ and changed the correct answer from ‘low fat’ to ‘high fat’. This allows for more consistent interpretation because cheddar cheese (even when reduced fat) has more than 3 g of fat per 100 g. Since correct options are based on % (per 100 g of the food), we also omitted portions from response options. This is Q2.5.1 in the NSKQ and Q3 in the ANSKQ.
- ***Item 2.2*** on high/low carbohydrate foods was adapted from existing questionnaires. The correct answers had at least 20 g of carbohydrate, which is about the amount of carbohydrate in a ‘serving’ of grain foods [10]. However, the correct answers did not factor in definitions for ‘high carbohydrate’ stated by on European Food Safety Authority (EFSA)/Food Standards Australia New Zealand (FSANZ) [8, 9] (‘*not less than 90 % of average energy derived as carbohydrate* and *more than 15% by weight is carbohydrate*’). Twenty grams of carbohydrate is a small amount in absolute terms. Therefore, item 2.2 has been modified from ‘*Do you think these foods are high/low in carbohydrate*’ to ‘*Which of these foods would provide enough carbohydrate for a recovery from about 1 hour of high intensity aerobic exercise? Assume the athlete weighs about 70 kg and has an important training session again tomorrow*’. The correct options have been adjusted accordingly. These are Q12, 13 in the ANSKQ.
- ***Item 2.9*** on high/low protein foods was adapted from existing questionnaires. Quinoa was included as an incorrect option because although this grain is relatively high in protein compared to other grains, its absolute protein content is still quite low. In most countries foods can be classified as a good source of protein if they have at least 10 g of protein per serving or as ‘high’ in protein if at least 20% of energy content is protein [8, 9]. Item 2.9 was modified to ask if foods contain enough protein to promote muscle growth after a bout of resistance exercise (with > 20 g being considered an acceptable dose of protein). These are Q14, 15, 16 in the ANSKQ.
- ***Item 2.6.4*** previously stated ‘*protein absorption in a single sitting is limited*’. Here, we were referring to the ability of the body to synthesise muscle from ingested protein. To clarify this, the statement was modified to ‘*The body has a limited ability to use protein for muscle synthesis*’. This is Q6 in the ANSKQ.
- ***Item 3.2*** asked about ‘*best sources*’ of micronutrients. Best is a comparative term and depends on whether you are referring to the amount of nutrient per 100 g or per serving. Therefore, these items were modified to ask if the foods were ‘*good sources*’ of each nutrient.
  Correct answers were based on EFSA/ FSANZ criteria, which state that for a food to be considered a ‘good’ source, it must meet 30% of the Recommended Dietary Intake (RDI) [8, 9]. These are now Q3.1.6 to 3.1.8 in the NSKQ and are not included in the ANSKQ.
- ***Item 3.3.5*** *‘Athletes have increased magnesium losses in sweat’* was deleted due to a lack of consensus.
- ***Item 4.3*** on optimal carbohydrate concentration for hydration purposes (i.e. in sports drink) was omitted because this is a confusing issue. Carbohydrate supports performance, not hydration; at low concentrations (~ 3%) absorption and retention is likely similar or mildly better than water. At higher concentrations (e.g. 8%) fluid absorption and retention may be impaired.
- ***Item 5.5*** was on supplements that are supported for *‘improving sporting performance’*. Leucine may have positive effects on muscle synthesis but not necessarily performance per se. This question was re-worded to ask which supplements were proven in relation to ‘*improving sporting performance and/or body composition’*. This is Q34 in the ANSKQ.

Items modified due to change in evidence/emerging themes

- Due to the prevalence of Vitamin D deficiency, the **item 3.1.5** ‘Vitamin D enhances calcium absorption’ was added and **item 3.2.4** ‘*Fatty fish is a good source of Vitamin D’* replaced ‘*Milk, yoghurt and Cheese are the best sources of magnesium*’. These questions are not included in the ANSKQ.
- ISSN consensus statement recommends 1.4–2.0 g protein/kg/day. For ***item 2.7*** on protein needs of well-trained athletes, the correct option was changed from 130 g to 150 g, to reflect higher recommendations. Also, 250 g was deleted as an ‘incorrect answer’, given evidence supporting potential benefits of very high protein intakes (2.3–3.1 g/kg) for retention of lean muscle mass for athletes on hypocaloric diets [6]. This is Q21 in the ANSKQ.
- For ***Item 5.6*** the correct option for banned substances was changed from ‘*glycerol*’ to ‘*testosterone*’. This is Q35 in the ANSQ.

Re-wording of several items has also been undertaken to make language simpler. For example:

- ***Item 1.2.2*** was re-worded from ‘*Increasing protein in the diet is the main dietary change needed when only muscle gain is desired*’ to ‘*Eating more protein is the most important dietary change if you want to have more muscle*’. This is Q17 in the ANSKQ.
- ***Item 6.5.2*** was re-worded from ‘Drinking large amounts of alcohol can reduce recovery from injury’ to ‘Drinking lots of alcohol can make it harder to recover from injury’. This question is not included in the ANSKQ.
- A copy of the new tools are available from Dr. Gina Trakman (g.trakman@latrobe.edu.au)
- The Italian ANSKQ can be obtain from: Umberto Placentino (uplacentino@gmail.com). For additional work on the tools in Italian contact Michele Gobbi and Roberto Cattivelli (michelegobbi93@icloud.com; roberto79cattivelli@gmail.com)
- The Swedish NSKQ can be obtained from: Lilja Guðmundsdóttir (lig31@hi.is)
- The Turkish NSKQ can be obtained from: Onur Çırak (ocirak@ankara.edu.tr)
- The Chinese NSKQ can be obtained from: Jackie Shao (utaipei101@gmail.com)
- The Malay NSKQ can be obtained from: Norashikin Binti Mustafa (p95560@siswa.ukm.edu.my)
- Details on the German NSKQ will be available shortly.

Many thanks,

Gina Trakman, Freddy Brown, Adrienne Forsyth and Regina Belski.

## Data Availability

The data used/and or analyses during the current study are available from the corresponding author on reasonable request.

## Abbreviations

ANSKQ: Abridged nutrition for sport knowledge questionnaire
EFSA: European Food on European Food Safety Authority
FSANZ: Food Standards Australia New Zealand
ISSN: International society of sports nutrition
NSKQ: Nutrition for sport knowledge questionnaire
WADA: World Anti-Doping Agency
